# Impact of Co/Ni Ratio on Solidification Characteristics and As-Cast Microstructure of Co-Al-W-Based Superalloys

**DOI:** 10.3390/ma19132843

**Published:** 2026-07-03

**Authors:** Sifan Yu, Minqing Wang, Nan Jiang, Xiaopeng Xu

**Affiliations:** 1 Central Iron and Steel Research Institute, Beijing 100081, China; yusifan@126.com (S.Y.);; 2 Gaona Aero Material Co., Ltd., Beijing 100081, China

**Keywords:** Co-Al-W-based superalloy, Co/Ni ratio, as-cast microstructure, solidification behavior, dendritic segregation

## Abstract

This study systematically investigated the effects of Co/Ni ratios (0.6–2.0) on the solidification behavior, as-cast microstructure, and element segregation of Co-Al-W-based superalloys, and elucidated the mechanism of thermodynamic and kinetic synergistic regulation. The results show that increasing the Co/Ni ratio has a negligible effect on the liquidus and solidus temperatures, but it significantly lowers the dissolution temperature of the γ′ phase, thereby expanding the alloy’s heat treatment window (HTW) from 215 °C to 269 °C. As the Co/Ni ratio increased from 0.6 to 2, the SDAS at the center of the alloy ingot decreased from 112.4 μm to 43.3 μm, resulting in a significant refinement of the as-cast microstructure. The dendritic segregation coefficients for positively segregating elements such as Ta, Hf, and Al, as well as negatively segregating elements such as W, all approached 1 significantly, effectively suppressing microsegregation during solidification. This study reveals the multidimensional synergistic regulation mechanism of the Co/Ni ratio on the non-equilibrium solidification behavior of highly alloyed Co-Al-W-based superalloys and quantitatively elucidates the relationship between the Co/Ni ratio, the microstructural uniformity of as-cast specimens, and the heat treatment process window. For the first time in a highly alloyed multi-component Co-Al-W system, a correlation has been established between the Co/Ni ratio, element segregation, dendrite coarsening coefficient, and heat treatment window.

## 1. Introduction

Cobalt-based superalloys possess a high initial melting point, excellent thermal corrosion resistance, fatigue resistance, and good weldability [1,2]. They represent ideal candidate materials with significant application potential for hot-end components in aerospace engines and gas turbine systems [3]. Conventional strengthening mechanisms for cobalt-based superalloys primarily involve solution strengthening and carbide strengthening, resulting in maximum service temperatures significantly lower than those achievable with nickel-based superalloys strengthened by precipitation of secondary phases. In 1971, Ch. S. Lee et al. [4] first discovered during their study of Co-Al-W ternary alloys that tungsten addition induces the formation of an ordered FCC superlattice coherent precipitation phase. This breakthrough paved the way for the development of novel cobalt-based superalloys. Later, in 2006, J. Sato’s team [5] identified this coherent precipitation phase in the ordered FCC superlattice as L1_2_—Co_3_(Al,W). This discovery made it possible to solve the problem of hardening conventional cobalt alloys and laid the foundation for the development of new cobalt-based superalloys hardened by the γ′ phase.

It should be noted that the γ′ phase in Co-Al-W-based superalloys has a narrow thermodynamic stability range and tends to decompose at high temperatures [6,7]. To address this issue, many researchers have conducted in-depth studies on the optimization of multi-component Co-Al-W-based superalloys: Shinagawa K [8] et al. studied Co-Ni-Al-W alloys and found that both the width of the single-phase γ′ region and the width of the two-phase γ/γ′ region increased significantly when the Ni content was increased from 10 at.% to 30 at.%. Simultaneously, Ni addition improved the alloy’s mechanical, creep, and oxidation properties [9,10,11,12]. Katsushi et al. [13] found that adding Ta and Ni to Co-Al-W-based alloys elevated the γ′ phase dissolution temperature to 1100 °C, substantially enhancing γ′ phase stability; Chung D W et al. demonstrated [14] that adding just 4 at.% Cr to Co-Al-W-based alloys significantly improved oxidation resistance. Therefore, to enhance the γ’ phase dissolution temperature, microstructural stability, high-temperature mechanical properties, and oxidation resistance of Co-Al-W-based superalloys, researchers are continuously increasing the alloying complexity of these materials. Consequently, ternary Co-Al-W alloys have gradually evolved toward multi-component alloys [15,16,17,18].

However, most existing research on Co-Al-W-based superalloys has focused on the microstructure and mechanical properties of the alloys after heat treatment. Systematic reports on the decisive role of non-equilibrium solidification behavior in determining the as-cast microstructure, as well as the synergistic regulatory mechanisms of the solidification process across a wide range of Co/Ni ratios, remain scarce [19,20,21,22,23,24,25,26,27,28,29,30,31,32,33,34,35]. The dendritic morphology, element segregation, and characteristic solidification temperatures during casting directly determine the homogenization efficiency of subsequent solution treatment, the dissolution of harmful TCP phases, and the upper limit of the alloy’s ultimate service performance. Currently, there are few systematic reports on the synergistic regulation of characteristic solidification temperatures, dendritic morphology evolution, and element segregation behavior in highly alloyed, multi-component Co-Al-W-based superalloys with respect to the Co/Ni ratio.

Based on this, this study designed four experimental alloys with Co/Ni ratios ranging from 0.6 to 2.0 within the Co-Ni-Al-W multi-component superalloy system. Alloy ingots were prepared via vacuum induction melting. The study systematically investigated the effects of the Co/Ni ratio on the alloy’s characteristic solidification temperature, the evolution of as-cast dendrite morphology, and element segregation behavior during solidification, elucidating the underlying thermodynamic and kinetic synergistic regulation mechanisms. This study not only fills a gap in research on the control of solidification behavior in multi-component Co-Al-W-based superalloys but also provides systematic theoretical support and quantitative data for the composition design, casting process optimization, and development of subsequent heat treatment processes for new high-performance Co-Al-W-based superalloys.

## 2. Experimental Section

This study designed four groups of Co-Ni-based alloys with different Co/Ni ratios (0.6, 1.2, 1.5, and 2). Based on their respective Co/Ni ratios, they were designated as 0.6Co/Ni, 1.2Co/Ni, 1.5Co/Ni, and 2.0Co/Ni. All four alloys were fabricated into Φ80 mm × 200 mm ingots using a 25 kg vacuum induction melting furnace under strictly identical process conditions. The melting process was conducted under a vacuum level of 1.0 × 10^−2^ Pa. The molten alloys were maintained at a melting temperature of 1550 °C for a holding time of 20 min to ensure complete homogenization. High-purity graphite molds preheated to 800 °C were employed for casting. After pouring at 1500 °C, the ingots were naturally cooled to room temperature in the molds. The cooling rate in the mold exhibited a typical radial gradient distribution: it was highest at the edge region in direct contact with the mold wall, decreased gradually toward the R/2 position, and reached the minimum at the ingot center. The cooling rate at the same radial position was identical for all four alloys, and all characterization samples were taken from the same height to eliminate axial cooling rate variations. The designed and measured chemical compositions (wt.%) of the four alloys are listed in Table 1.

To observe the microstructural variations from center to edge throughout the entire four-alloy ingot, the microstructure of different regions was examined using an optical microscope (OM, Olympus GX71, Olympus Corporation, Tokyo, Japan). Prior to microstructural analysis, samples were ground with a grinding wheel to remove wire-cutting marks, followed by mechanical polishing with SiC sandpaper. Subsequently, samples underwent polishing, ultrasonic cleaning, rinsing, and drying. Chemical etching followed, with samples cleaned and air-dried post-etching. Etched specimens were examined under a metallographic microscope. The etching solution composition was HCl:H_2_O_2_:H_2_O = 1:1:2. Using ImageJ-Pro software, Version 1.53t, National Institutes of Health, Bethesda, MD, USA, the secondary dendrite arm spacing (SDAS) of the four alloys was measured by the linear intercept method. Transverse lines were drawn perpendicular to the secondary dendrite trunk. The number of secondary dendrite arms intersected by each line and the corresponding line length were counted to calculate the average spacing per dendrite arm. Three parallel specimens were taken from each sample. Five non-overlapping metallographic images were acquired for each specimen, with no fewer than 50 valid data points counted per image. The final results were expressed as the mean and standard deviation.

To characterize element segregation in the as-cast microstructure, the non-equilibrium solidification process of the alloy was simulated using the Scheil–Gulliver solidification model integrated within the J-MatPro software, Version 15.0, Sente Software Ltd., Guildford, UK, utilizing the Ni-based superalloys database. The input composition basis was set to the measured bulk chemical composition in weight percentage. The simulation considered the liquid phase, the gamma matrix phase, and the gamma prime precipitate phase. The applied Scheil–Gulliver model assumed complete solute mixing in the liquid phase and completely neglected back diffusion in the solid phase. Element distribution analysis was performed to determine the micro-area concentrations (at.%) on different regions within the central area of the same cross-section for four groups of alloys with varying Co/Ni ratios using EPMA (Shimadzu EPMA-1720, Shimadzu Corporation, Kyoto, Japan) operated at an accelerating voltage of 15 kV and a beam current of 20 nA. To ensure the accuracy of the test data, no less than 20 micro-areas with identical contrast were selected for point testing in both the dendrite cores DC and interdendritic ID regions, and the final results were expressed as mean values with standard deviations. Notably, the TCP phase precipitates in the interdendritic regions were strictly avoided during EPMA point testing, and only the γ matrix phase was measured, to ensure that the calculated segregation coefficients only reflect the element distribution characteristics of the matrix.

To determine the solidification characteristic temperatures and phase transformation temperatures of the alloys, a differential scanning calorimeter (DSC, STA-449C, Netzsch Gerätebau GmbH, Selb, Germany) was used to characterize the phase transformation behavior during continuous heating. Φ3 × 1 mm^2^ specimens were taken from the center of the ingots for DSC analysis. The central region was selected because it has the slowest cooling rate and the most severe microsegregation during solidification, which can best represent the intrinsic non-equilibrium solidification behavior of the alloy. After cleaning the specimen surfaces with #400 SiC sandpaper, specimens weighing approximately 20 mg were placed in high-purity alumina crucibles. The specimens were then heated in the DSC instrument under a vacuum of 1 × 10^−3^ Pa from room temperature to 1400 °C at a rate of 10 °C/min. The instrument was calibrated using pure standard metals and baseline correction was performed using an empty crucible. Three replicate measurements were conducted for each alloy to ensure reproducibility. The solidus and liquidus temperatures were identified from the melting endotherm. Specifically, the solidus temperature was determined by the extrapolated onset temperature, while the liquidus temperature was taken as the peak temperature. The dissolution temperature of the γ′ phase was determined from the peak temperature of its corresponding endothermic reaction. This heating rate is the standard rate for DSC characterization of superalloys, which balances phase transformation resolution and test efficiency.

## 3. Results

### 3.1. Effect of Co/Ni Ratio on Solidification Characteristic Temperature

**Figure 1:**
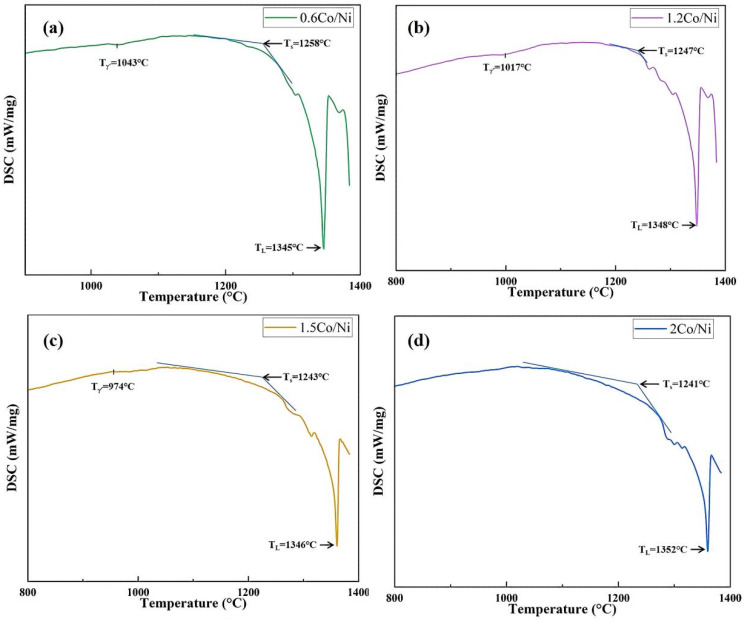
DSC heating curves and characteristic phase transition temperatures of alloys with different Co/Ni ratios: (**a**) 0.6Co/Ni; (**b**) 1.2Co/Ni; (**c**) 1.5Co/Ni; (**d**) 2.0Co/Ni.

In this study, vacuum differential scanning calorimetry (DSC) was used to systematically characterize the phase transformation behavior of four alloys with different Co/Ni ratios during continuous heating. The characteristic temperatures corresponding to the solid–liquid phase transition and the complete dissolution of the γ′ phase were precisely determined, and the influence of the Co/Ni ratio on the control of the alloy’s solidification temperature range and heat treatment window was quantitatively analyzed. The DSC heat flow curves obtained from the tests are shown in Figure 1, and the phase transition temperatures calibrated based on characteristic peaks are presented in Table 2. Based on these test results, this study identified five key parameters: liquidus temperature (*T*_L_), solidus temperature (*T*_s_), γ′ phase dissolution temperature (*T*_γ′_), heat treatment window (*HTW* = *T*_s_ − *T*_γ′_), and solidification range (Δ*T* = *T*_L_ − *T*_s_). It should be noted that the γ’ phase dissolution temperature (*T*_γ′_) is the critical temperature at which the γ’ phase completely resolves into the γ matrix during heating; it serves as the core basis for establishing the solution heat treatment process for superalloys. In this paper, the heat treatment window (*HTW*) is defined using the standard formula: *HTW* = *T*_s_ − *T*_γ′_ [37].

The results show that as the Co/Ni ratio increases from 0.6 to 2.0, *T*_L_ remains stable between 1345 and 1352 °C, and *T*_s_ remains stable between 1237 and 1258 °C, with no obvious monotonic trend in either case. This indicates that within this composition range, the Co/Ni ratio has a negligible effect on the alloy’s melting point. *T*_γ′_ decreased sharply with increasing Co/Ni ratio: *T*_γ′_ was 1043 °C for the 0.6 Co/Ni alloy, 1017 °C for the 1.2 Co/Ni alloy, and 974 °C for the 1.5 Co/Ni alloy. Notably, in the DSC curve of the 2.0Co/Ni alloy during heating, no distinct endothermic peak corresponding to the complete dissolution of the γ’ phase was observed. Since the changes in *T*_L_ and *T_s_* were both insignificant, the solidification temperature interval Δ*T* remained stable between 87 and 111 °C. At the same time, due to the significant decrease in *T*_γ′_, the heat treatment window (*HTW*) of the alloy widened significantly as the Co/Ni ratio increased, widening from 215 °C for the 0.6Co/Ni alloy to a maximum of 269 °C for the 1.5Co/Ni alloy.

### 3.2. Effect of Co/Ni Ratio on Secondary Dendrite Arm Spacing (SDAS)

**Figure 2:**
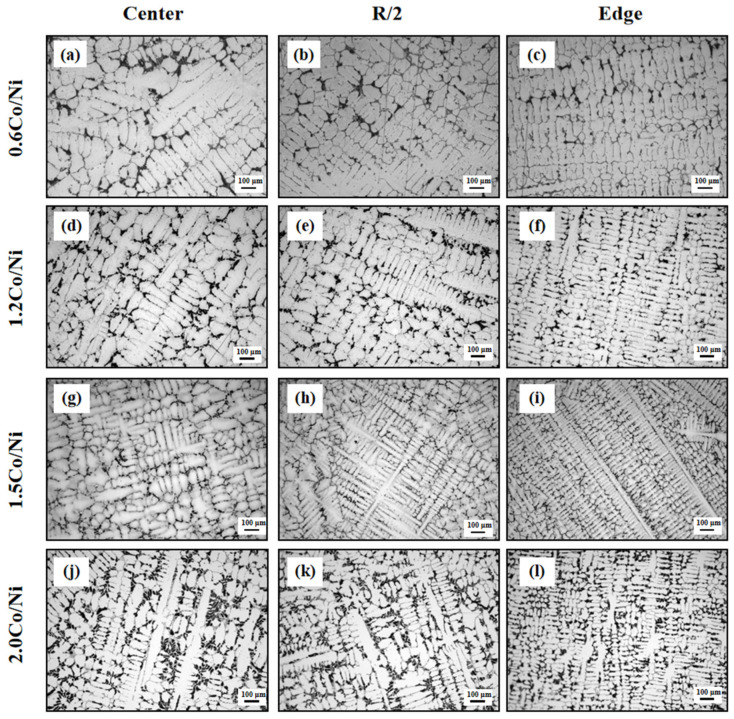
OM image showing the radial gradient of dendritic morphology (Center, R/2 position, and Edge) across ingots with varying Co/Ni ratios: (**a**–**c**) 0.6Co/Ni; (**d**–**f**) 1.2Co/Ni; (**g**–**i**) 1.5Co/Ni; (**j**–**l**) 2.0Co/Ni.

Figure 2 displays the typical metallographic microstructures at the center, R/2 position, and edge of four groups of alloys with different Co/Ni ratios (0.6Co/Ni to 2.0Co/Ni) in the as-cast state. As evident from Figure 2, all alloys exhibit typical and well-developed dendritic morphology, with a clear distinction between dendrite core (DC) and interdendritic (ID) regions. Within the same ingot, dendritic morphology shows a pronounced refinement trend from the center toward the edge.

**Figure 3:**
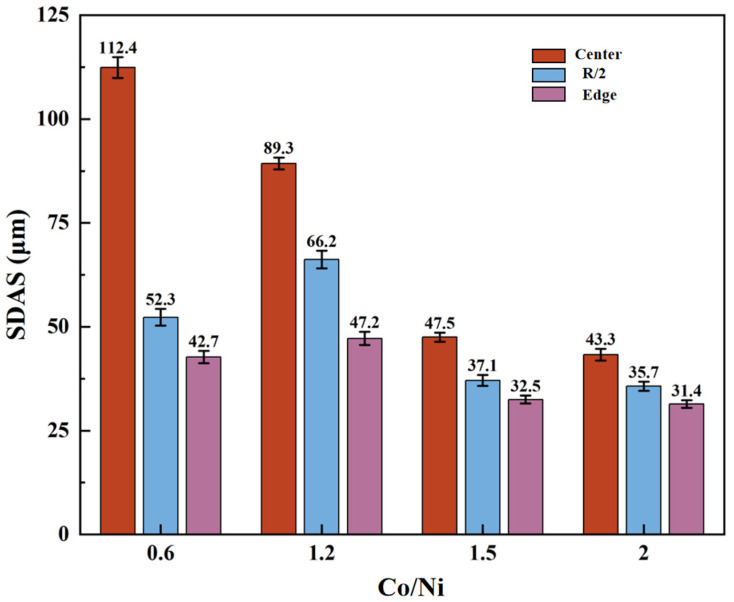
Variation Trends of SDAS (μm) in Different Regions of Alloys with Different Co/Ni Ratios.

To further investigate the influence of the Co/Ni ratio on solidification microstructure, this study employed the linear intercept method to statistically analyze the secondary dendrite arm spacing (SDAS, λ_2_) at different radial positions (center, R/2, and edge) of four alloy ingots. Three parallel specimens were taken from each sample, and the average value from five metallographic images per specimen was used as the final result, as shown in Table 3 and Figure 3.

The results show that the SDAS of all alloys exhibits a pronounced radial distribution that gradually decreases from the center to the edge of the ingot. In the example of the 0.6Co/Ni alloy, SDAS reaches a maximum value of 112.4 μm at the center, decreases to 52.3 μm at the R/2 position, and continues to decrease to 42.7 μm at the edge. It should be noted that changes in the Co/Ni ratio also have a significant influence on SDAS: With the increase in the Co/Ni ratio from 0.6 to 2.0, the center region shows a pronounced refinement, with the SDAS value decreasing significantly from 112.4 μm to 43.3 μm. Meanwhile, the R/2 and edge regions exhibit a non-monotonic change at lower Co/Ni ratios, followed by refinement at Co/Ni ratios greater than or equal to 1.5.

### 3.3. Effect of Co/Ni Ratio on Interdendritic Element Segregation

To quantitatively characterize the thermodynamic trend of alloying element distribution at the initial stage of solidification, a solid–liquid fractionation coefficient (*p*) was introduced. This coefficient is defined as the ratio between the concentration $C_{S}$ (at.%) of a specific alloying element in the solid phase and its concentration $C_{L}$ (at.%) in the liquid phase during the initial solidification phase [38] and can be calculated using Equation (1):

(1) $$p\;={\;C}_{S}/C_{L}$$

If p > 1, the elements tend to accumulate in the dendrite core; if p < 1, the elements tend to segregate in the interdendritic regions. To determine the characteristics of the initial distribution of elements in the system under study more accurately, the process of non-equilibrium solidification of the alloy was modeled using the Scheil–Gulliver model in the J-Matpro software (Version 15.0), and the initial partition coefficients of the solid and liquid phases were calculated and presented in Table 4. Results indicate that across four groups of alloys with different Co/Ni ratios, although the initial solid–liquid fractionation coefficients *p* for each alloying element exhibit slight fluctuations, their overall thermodynamic distribution tendencies remain stable. Specifically, the *p* values for Ta, Hf, and Al were all less than 1, indicating a positive segregation tendency, while the *p* values for W and Cr were greater than 1, indicating a negative segregation tendency. This theoretical simulation result indicates that in the Co-Ni-Al-W multicomponent system, changes in the Co/Ni ratio did not alter the thermodynamic driving force for the initial distribution of alloying elements between the solid and liquid phases.

**Figure 4:**
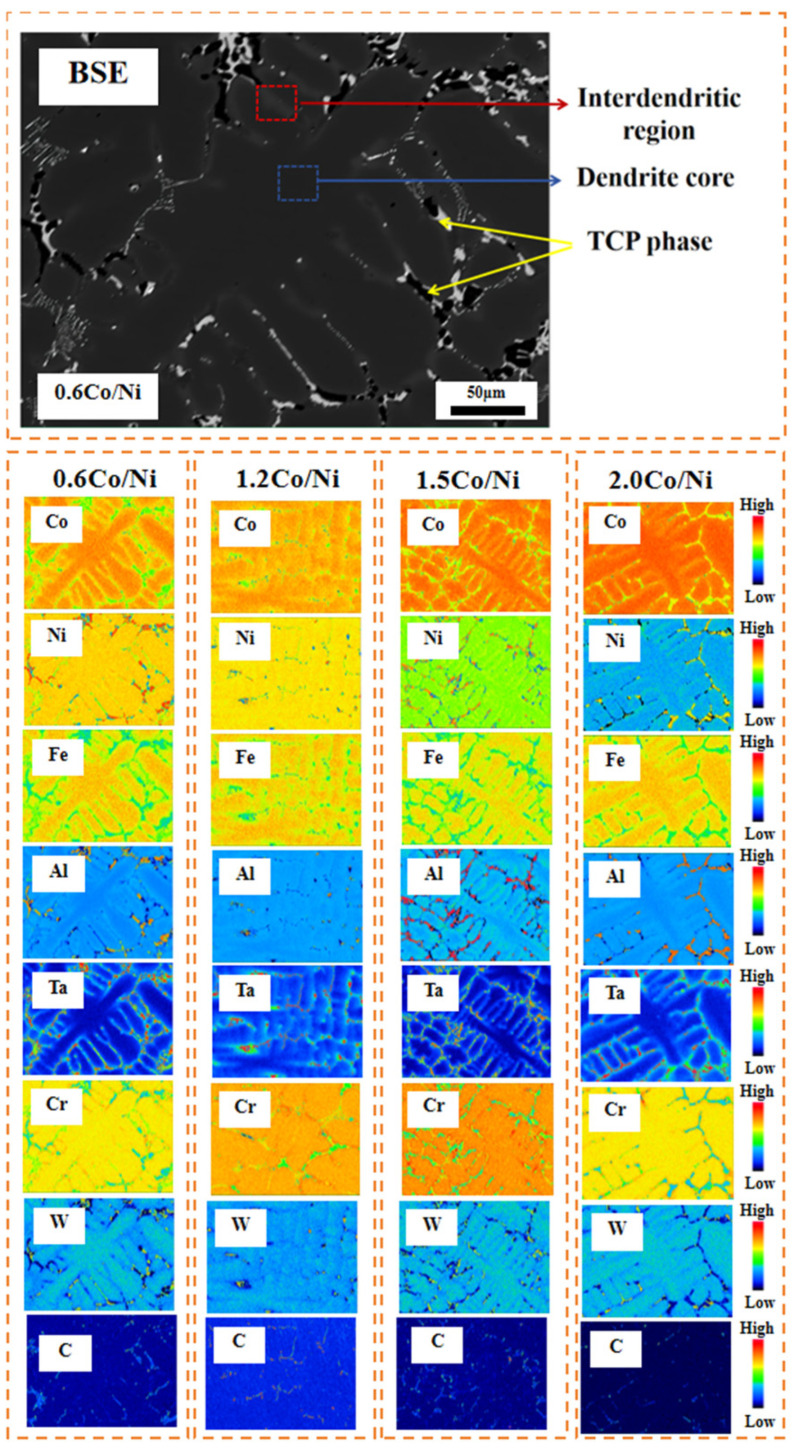
Element distribution in alloys with varying Co/Ni ratios. The enlarged BSE image of the 0.6Co/Ni alloy identifies key microstructural regions.

Using electron probe microanalysis EPMA technology, precise microarea composition measurements were performed on the DC and ID regions of four groups of as-cast alloy center samples, as shown in Figure 4. A representative backscattered electron BSE image was utilized to precisely identify the dendrite core, the interdendritic region, and the TCP phase precipitates. By observing the contrast differences in the EPMA elemental distribution maps, it was found that Al, Ta, and Hf can be regarded as positive segregation elements, tending to enrich in the ID region during solidification, with Ta and Hf exhibiting the strongest segregation. Conversely, Co and Fe can be considered negative segregation elements, tending to enrich in the DC region during solidification.

To quantitatively analyze the degree of micro-segregation of each element in the as-cast microstructure, the segregation coefficient *K*_i_ is introduced. This coefficient is defined as the ratio of the interdendritic region (ID) composition to the dendrite core (DC) composition and can be calculated using Equation (2):

(2) $$K_{i}=C_{ID}^{i}/C_{DC}^{i}$$

**Figure 5:**
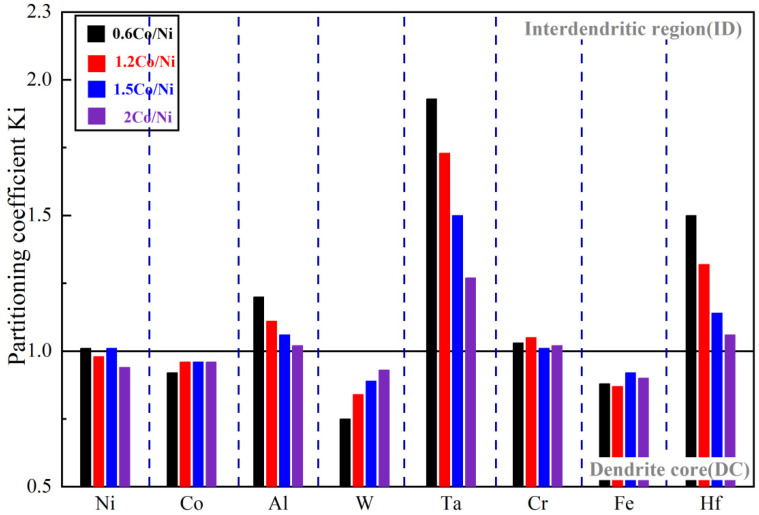
Dendritic Segregation Coefficient for Alloys with Different Co/Ni Ratios.

In the equation, $C_{ID}^{i}$ and $C_{DC}^{i}$ represent the atomic fractions (at.%) of element i in the interdendritic region (ID) and dendrite core (DC), respectively. When *K*_i_ > 1, the element exhibits positive segregation; when *K*_i_ < 1, it exhibits negative segregation. The greater the deviation of *K*_i_ from 1, the more pronounced the segregation of that element. The specific *K*_i_ values calculated via EPMA point measurements are detailed in Table 5, and their variation with the Co/Ni ratio is shown in Figure 5.

The results indicate that as the Co/Ni ratio increased from 0.6 to 2.0, the segregation coefficients *K*_i_ of positively segregating elements Al, Ta, and Hf exhibited a significant decreasing trend, gradually approaching 1. Specifically, the *K*_i_ of Ta decreased from 1.93 to 1.27, the *K*_i_ of Hf decreased from 1.50 to 1.06, and the *K*_i_ of Al decreased from 1.20 to 1.02. The *K*_i_ values of negatively segregating element W exhibited a marked upward trend, rising from 0.75 in the 0.6Co/Ni alloy to 0.93 in the 2.0Co/Ni alloy, also gradually approaching 1. The *K*_i_ of Ni, Cr, and Fe showed no significant fluctuation with Co/Ni ratio changes, consistently remaining within the range of 0.87 to 1.05, indicating weak segregation tendency. The quantitative analysis results collectively demonstrate that the degree of segregation of both positively segregating elements (Ta, Hf, Al) that tend to enrich in the interdendritic regions and the negatively segregating element (W) that tends to enrich in the dendrite cores was effectively alleviated with the increase in the Co/Ni ratio. This result confirms that despite the constant initial thermodynamic partitioning driving force (i.e., the equilibrium partition coefficient *p*) of each alloying element, a high Co/Ni ratio significantly enhances the chemical homogeneity of the final as-cast microstructure.

## 4. Discussion

### 4.1. Thermodynamic Effects of Co/Ni Ratio on Solidification Behavior and Heat Treatment Window

This study has shown that increasing the Co/Ni ratio has a negligible effect on the liquidus temperature (*T*_L_) and solidus temperature (*T*_s_). This pattern is consistent with the findings of Zenk et al. [12] and Zhang et al. [20] in similar alloy systems. The main reason for this is that the atomic radius of Ni is 0.1246 nm and that of Co is 0.1251 nm. These two elements have similar atomic radii and form an infinite solid solution in the Ni-Co binary phase diagram [38]. Additionally, their melting temperatures differ only slightly ($T_{m}^{\mathrm{Ni}}$= 1455 °C; $T_{m}^{\mathrm{Co}}$= 1495 °C). Consequently, replacing Ni with Co in multicomponent superalloys does not generally result in a significant change in the crystal lattice binding energy, which allows the stability of the liquidus and solidus lines to be maintained. It should be noted that research conducted to date has mainly focused on low-alloyed ternary or quaternary Co-Al-W systems. This study confirms that in complex multicomponent systems containing alloying elements such as Cr, Ta, and Hf, a change in the Co/Ni ratio still has no significant effect on the melting characteristics of the alloy. This finding provides crucial thermodynamic evidence for the composition gradient design and stable casting process window of alloys in this system.

An increase in the Co/Ni ratio significantly lowers the γ’ phase dissolution temperature (*T*_γ′_): the *T*_γ′_ of the 0.6Co/Ni alloy is 1043 °C, while that of the 1.5Co/Ni alloy drops to 974 °C; in contrast, no distinct endothermic peak corresponding to the complete dissolution of the γ’ phase was detected in the DSC test of the 2.0Co/Ni alloy. It should be noted that previous studies by our team on this Co-Ni-based alloy system have confirmed via SEM, XRD, and TEM characterization that a stable L1_2_-type γ′ precipitate phase is present in the as-cast microstructure even at a high Co/Ni ratio of 2.0 [36]. Therefore, the absence of a characteristic peak in the DSC analysis does not indicate the absence of the γ′ phase. According to the study by Shinagawa et al. [8], the thermodynamic stability of the L1_2_-Ni_3_Al phase is significantly higher than that of the L1_2_-Co_3_(Al,W) phase. Furthermore, as the Co concentration increases, the Gibbs free energy of the γ’ phase rises, and its thermodynamic stability decreases, resulting in its precipitation only at lower temperatures. The findings of Zenk et al. [39] on nine-component Co-Ni-based superalloys indicate that Co is primarily distributed in the γ matrix, where it acts to stabilize the γ matrix, while Ni is primarily distributed in the γ’ phase. Therefore, when the Co/Ni ratio in the alloy increases, the amount of Co in the γ matrix increases, while the amount of Ni available for forming the γ’ precipitation phase decreases. This compositional change directly reduces the chemical potential of the elements forming the γ’ phase. Furthermore, studies have shown that as the Co content increases, the solubility of Al and Ta in the γ’ phase also decreases [40]. This weakens the thermodynamic stability of the γ’ phase at high temperatures, meaning that the γ’ phase can only reach the supersaturation conditions required for precipitation at lower temperatures. Second, based on the d-electron alloy design theory proposed by Morinaga et al. [41], the phase stability of superalloys is strongly correlated with the average d-orbital energy level ($\bar{Md}$) of the alloy matrix. Since Co possesses a higher Md value than Ni (Md_Co_ = 0.777 eV, Md_Ni_ = 0.717 eV), an increased Co/Ni ratio inevitably elevates the $\bar{Md}$ of the γ matrix. According to this theory, to maintain the thermodynamic structural stability of the FCC matrix, it tends to reject solute elements with excessively large *Md* values, such as Ta (Md_Ta_ = 2.224 eV). This electronic repulsion effect significantly alters the elemental partitioning between the γ and γ’ phases, directly reducing the chemical potential required for γ’ formation, which further reduces the nucleation driving force and macroscopically lowers the dissolution temperature of the γ’ phase.

It is worth noting that the intensity of the DSC peak characteristic of the γ’ phase essentially depends on the concentration and absolute value of the heat flux changes during the phase transition, which are directly related to the size and nucleation-growth behavior of the γ’ phase. The study by Mallikarjuna et al. [42] clearly indicates that the characteristic endothermic peaks of the γ’ phase dissolution are highly dependent on the size of the precipitates: for large γ’ phase precipitates with a size of 940 nm, the activation energy for dissolution reaches as high as 377 kJ/mol, and the phase transition occurs within a narrow temperature range, resulting in a sharp DSC peak. In contrast, the ultrafine γ’ phase with a particle size of 27 nm has a dissolution activation energy of only 242 kJ/mol. The phase transition responds more gradually to temperature changes, and the latent heat of phase transition is dispersed over an extremely wide temperature range, making it impossible to form a sharp characteristic peak that can be detected by DSC equipment. In our group’s previous research, an increase in the Co/Ni ratio caused the size of the γ’ phase to decrease from 147.49 nm to 40.28 nm [36]. Consequently, the formation of this ultrafine γ’ phase causes its dissolution during continuous DSC heating not to occur concentrated within a narrow temperature range, but rather to dissolve continuously over a wide temperature range from room temperature to below the solidus. The latent heat of phase transition is completely dispersed, and the rate of change in the heat flow signal falls below the detection sensitivity of the DSC instrument, ultimately resulting in the disappearance of the characteristic peak.

The broadening of the heat treatment window represents the thermodynamic change with the greatest engineering value in this study. For highly alloyed Co-Al-W superalloys, achieving compositional homogenization in the as-cast microstructure requires prolonged solution treatment at temperatures close to the solidus line. However, an excessively narrow heat treatment window can easily lead to initial melting of the alloy, thereby limiting the ability to increase the solution treatment temperature and reducing homogenization efficiency. In this study, when the Co/Ni ratio was increased from 0.6 to 1.5, the heat treatment window widened from 215 °C to 269 °C. This means that the alloy can be subjected to higher solution treatment temperatures without experiencing initial melting, thereby accelerating the homogenization and diffusion of refractory elements. This provides a wider margin for error and greater process flexibility in the formulation of subsequent heat treatment processes [20].

### 4.2. Mechanisms Governing the Effect of Co/Ni Ratio on Solidification Segregation Behavior

In this study, the simulation results from the Scheil–Gulliver model (Table 4) indicate that adjusting the Co/Ni ratio did not alter the positive or negative nature of the initial solid–liquid partition coefficients (*p*) for elements such as Al, Ta, Hf, and W; that is, the thermodynamic partition tendencies of these elements remained stable. However, EPMA measurements reveal that as the Co/Ni ratio increases from 0.6 to 2.0, the final segregation coefficients (*K*_i_) for the positively segregating elements Ta, Hf, and Al, as well as the negatively segregating element W, all show a clear tendency toward 1. This result indicates that the improvement in the degree of dendritic segregation in the alloy due to a high Co/Ni ratio is not attributable to changes in thermodynamic segregation tendencies, but rather results from the combined effects of accelerated diffusion kinetics and secondary phase transformation behavior.

Dendritic segregation during non-equilibrium solidification essentially results from the inability of solute atoms to achieve uniform distribution via diffusion following solute redistribution at the solid–liquid interface. In this study, the increase in Co content resulting from a higher Co/Ni ratio is the primary factor driving the significant enhancement of solute diffusion kinetics; this principle has been extensively confirmed and quantitatively verified in studies of nickel-based and cobalt-based superalloys. Co itself possesses an intrinsic diffusion ability in the γ matrix and the liquid phase of the alloy that is far greater than that of refractory elements such as W, Ta, and Hf. An increase in Co content significantly reduces the activation energy for the diffusion of refractory elements between the liquid and solid phases of the alloy, substantially increasing their mutual diffusion coefficients. Zhang et al. [20] demonstrated, through rigorous diffusion pair experiments and quantitative fitting, that an increase in the Co content can enhance the diffusion coefficients of refractory elements such as W in the γ matrix of nickel-cobalt-based alloys. They also elucidated the underlying mechanisms by which Co reduces elemental diffusion barriers, weakens the lattice binding energy of solute atoms, and promotes atomic migration. This diffusion acceleration mechanism also applies to the alloy system studied here: since the temperature range during the alloy solidification process is significantly higher than the temperatures at which diffusion coefficients have been previously measured, the diffusion acceleration effect of Co on refractory elements is expected to be even more pronounced. The suggested increase in the diffusion coefficient may fundamentally alter the solute distribution during solidification: W atoms enriched on the dendrite stems can be expected to rapidly diffuse into the interdendritic regions, while Ta, Hf, and Al atoms enriched in the interdendritic regions can be expected to accelerate their reverse diffusion toward the dendrite stems. This substantially weakens the solute concentration gradient at the solid–liquid interface, ultimately causing the element segregation coefficient Ki in the as-cast matrix to approach 1 significantly. This core mechanism is fully consistent with the conclusion reached by Pan et al. [37] in advanced nickel-based single-crystal superalloys, namely that Co-accelerated diffusion of refractory elements is the primary mechanism suppressing dendritic segregation.

Previous research by our team has demonstrated [36] that as the Co/Ni ratio increases from 0.6 to 2.0, the volume fraction of the Laves phase in the interdendritic regions of the as-cast alloy rises from 1.2% to 4.7%. This Laves phase is a typical TCP phase and has been rigorously identified via XRD and TEM characterization in our previous related work. Furthermore, this previous study confirmed that the TCP phase exhibits significant enrichment of segregating elements such as Ta, Al, and W. In the current study, the typical distribution of these TCP phase precipitates within the interdendritic regions is directly illustrated by the BSE image in Figure 4. It should be specifically noted that in this study, EPMA spot testing strictly avoided TCP phase precipitates in the interdendritic regions, focusing solely on compositional analysis of the γ matrix phase. Consequently, the measured Ki values were lower, accurately reflecting the reduced degree of element segregation in the matrix. Toward the end of solidification, solute elements enriched in the interdendritic regions are largely consumed through the formation of the TCP phase, further reducing the solute concentration in the final solidified matrix. This serves as a contributing factor to the improved compositional homogeneity of the matrix. Concurrently, the widening of the heat treatment window resulting from the increased Co/Ni ratio in this study provides sufficient temperature range for the complete dissolution of the TCP phase during subsequent solution treatment, enabling synergistic optimization of both the suppression of as-cast segregation and the elimination of harmful phases through heat treatment.

### 4.3. Synergistic Control of Dendrite Morphology by Solidification Heat Transfer Conditions and Solute Redistribution

Since all alloys were prepared under strictly identical process conditions, the effect of cooling rate has been fully eliminated, and the Co/Ni ratio was the only independent variable in this study. The SDAS of all four alloy ingots exhibited a distinct radial gradient distribution characterized by “smaller values at the edges and larger values at the center” (Figure 2), which is essentially attributed to spatial variations in macroscopic heat transfer conditions during solidification. The growth and coarsening behavior of secondary dendrites can be quantitatively described using the classical dendrite coarsening theory proposed by Kurz et al. [43]. This theory establishes a quantitative relationship between the inter-arm spacing of secondary dendrites and key solidification parameters, as shown in Equation (3); meanwhile, the core determinants of the local solidification time $t_{f}$ can be further clarified through the fundamental solidification heat transfer equation, as shown in Equation (4):

(3) $$\lambda_{2}\;=\;5.5{\left(A\text{⋅}t_{f}\right)}^{1/3}$$

(4) $$t_{f}=\frac{\Delta T}{G\cdot R}$$

In the equation, $\lambda_{2}$ represents the inter-dendrite spacing, A is the dendrite coarsening coefficient, $t_{f}$ is the local solidification time, Δ*T* is the solidification temperature range of the alloy, G is the temperature gradient at the solid–liquid interface, and R is the solidification rate. Within the same ingot, because the edges are in direct contact with the mold walls, the cooling rate is high and the residence time $t_{f}$ within the solidification temperature range is extremely short, which inhibits the diffusion of solute atoms and thus preserves a fine dendritic morphology. In contrast, the central region experiences significant heat accumulation, resulting in a substantially prolonged $t_{f}$, which allows the dendrites ample time to coalesce and coarsen, ultimately forming a dendritic structure with fine morphology at the edge and coarse morphology at the center. This pattern is fully consistent with the classical solidification theory of nickel-based/cobalt-based superalloys [44].

The most significant finding of this study is that, at the same radial position under identical macroscopic heat transfer conditions, an increase in the Co/Ni ratio still leads to a significant refinement of the SDAS, with the most pronounced refinement observed in the central region of the ingot. The SDAS decreased from 112.4 μm in the 0.6Co/Ni alloy to 43.3 μm in the 2.0Co/Ni alloy, representing a refinement of 61.5%. Furthermore, since the four alloys were prepared under identical melting, pouring, mold preheating, and cooling conditions, the influence of variations in macroscopic heat transfer conditions on the SDAS can be ruled out. At the same time, based on the DSC experimental results (Table 2), the solidification temperature range Δ*T* for the four alloys remained stable between 87 and 111 °C, with a maximum variation of only 24 °C. This corresponds to a theoretical variation of only 22% in the local solidification time $t_{f}$ at the same radial position. According to calculations using the Kurz formula [43], the theoretical impact on SDAS is only 7%, which is far lower than the measured refinement rate of 61.5%. Thus, it can be preliminarily concluded that the reduction in the apparent dendrite coarsening parameter *A* resulting from an increased Co/Ni ratio is a major contributing factor behind the significant refinement of the SDAS.

To quantitatively verify the above conclusions, this study performed normalized calculations of the apparent dendrite coarsening parameter *A* for the four alloys based on Kurz’s classical formula. Since the preparation and cooling conditions of the four alloys were completely uniform, the temperature gradient G at the solid–liquid interface at the same radial position on the ingot is a constant. Therefore, substituting $t_{f}\;=\;\Delta T/G$ into the Kurz formula yields the relative ratio of *A* between different alloys, as shown in Equation (5):

(5) $$\frac{A_{n}}{A_{0}}=\frac{\lambda_{2,n}^{3}/\Delta T_{n}}{\lambda_{2,0}^{3}/\Delta T_{0}}$$

In the equation, $A_{0}$, $\lambda_{2,0}^{3}$ and $\Delta T_{0}$ are the corresponding parameters for the 0.6 Co/Ni alloy, while $A_{n}$, $\lambda_{2,n}$ and $\Delta T_{n}$ are the corresponding parameters for alloys with different Co/Ni ratios. Taking the central region of the ingot as an example, the calculated relative A values are shown in Table 6.

The calculation results clearly show that as the Co/Ni ratio increases from 0.6 to 2.0, the relative value of the apparent dendrite coarsening parameter *A* decreases by as much as 95.5%. Its theoretical contribution to SDAS is $\sqrt[{3}]{0.045}-1\;\approx -64\%$, which closely matches the measured SDAS refinement of 61.5%. This result suggests that the significant reduction in the apparent dendrite coarsening parameter A caused by an increase in the Co/Ni ratio is the major contributing factor for SDAS refinement, and that fluctuations in local solidification time have a negligible effect on SDAS.

In their study of the solidification behavior of superalloys, Tai et al. demonstrated that, based on the Furer–Wunderlin model, the core physical significance of the apparent dendrite coarsening parameter *A* lies in its characterization of the driving force behind the processes of dendrite maturation and coalescence-induced coarsening; its magnitude is positively correlated with the degree of solute enrichment at the solid–liquid interface [44]: The more severe the solute enrichment at the solid–liquid interface, the greater the compositional gradient between dendrites and their stems, the stronger the driving force for dendrite coarsening, the larger the *A* value, and the more likely secondary dendrites are to undergo lateral growth and maturation-induced coalescence. In conjunction with the results of this study, simulations using the Scheil–Gulliver model show that as the Co/Ni ratio increases, the equilibrium solid–liquid partition coefficients (*p*) of major elements such as Al, Ta, Hf, and W exhibit only slight fluctuations and no significant monotonic changes, indicating that the Co/Ni ratio does not alter the thermodynamic partition tendencies of these elements. However, EPMA measurements indicate that increasing the Co/Ni ratio significantly reduces the degree of solute enrichment at the solid–liquid interface; this effect stems from the significant regulatory role of Co on solute diffusion kinetics. Zhang et al. demonstrated through diffusion pair experiments and quantitative fitting [20] that an increase in Co content can significantly reduce the activation energy for the diffusion of refractory elements such as W and Ta in Co-Ni-based superalloys, resulting in a substantial increase in the interdiffusion coefficients. This suggests that solute atoms accumulated between dendrites during solidification can be expected to rapidly diffuse in the opposite direction, resulting in a significant reduction in the solute concentration gradient at the solid–liquid interface. This finding is corroborated by EPMA measurements, which show that the Co/Ni ratio increased from 0.6 to 2.0, and the dendrite segregation coefficients for positively segregated elements such as Ta, Hf, and Al, as well as negatively segregated elements such as W, all significantly approached 1. Meanwhile, classical solidification theory studies by Kurz et al. indicate that a reduction in solute enrichment at the solid–liquid interface significantly weakens compositional undercooling, thereby inhibiting the lateral growth and maturation-induced coalescence of secondary dendrites [43], which further enhances the dendrite refinement effect in alloys with high Co/Ni ratios. Pan et al. reached a consistent conclusion in their study of advanced nickel-based single-crystal superalloys, demonstrating that an increase in Co content can achieve significant refinement of the as-cast dendritic microstructure by suppressing element segregation and reducing compositional undercooling [37].

**Figure 6:**
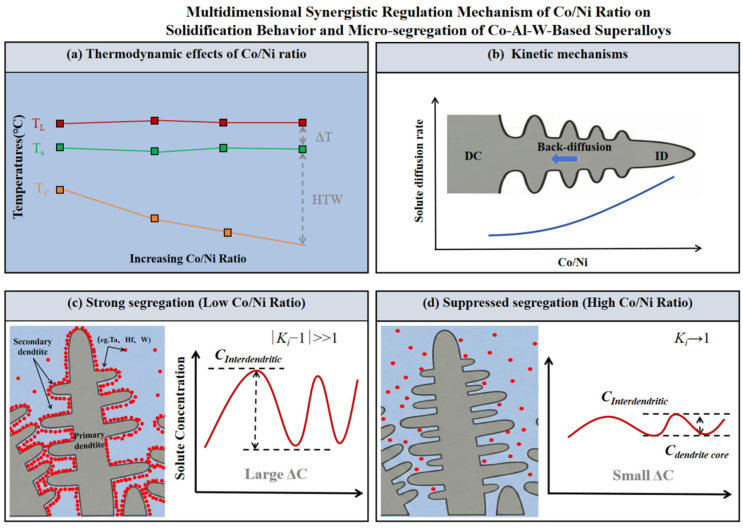
Schematic diagram illustrating the multidimensional synergistic evolution mechanism of solidification characteristics and microstructural segregation in alloys with different Co/Ni ratios.

Based on the above analysis, this study proposes a multidimensional synergistic control mechanism of the Co/Ni ratio on the solidification behavior of Co-Al-W-based superalloys, as shown in Figure 6. First, an increase in the Co/Ni ratio has no significant effect on the liquidus and solidus temperatures of the alloy, and does not alter the alloy’s solidification range or casting process window; however, it significantly lowers the dissolution temperature of the γ′ phase, thereby substantially broadening the alloy’s heat treatment window from a thermodynamic perspective and providing a wider temperature range for subsequent homogenization heat treatment of the as-cast microstructure. Simultaneously, an increased Co/Ni ratio significantly accelerates the diffusion kinetics of refractory solute atoms such as W, Ta, and Hf during solidification. Combined with the consumption of interdendritic segregated elements by the precipitation of the TCP phase during the late solidification stage, effectively suppressing the dendritic segregation of positively segregating elements such as Al, Ta, and Hf, as well as negatively segregating elements such as W. This causes the segregation coefficients in the as-cast matrix to approach 1 significantly, substantially improving the chemical homogeneity of the as-cast microstructure. This significant suppression of element segregation directly reduces the degree of solute enrichment and compositional undercooling at the solid–liquid interface, serving as the major contributing factor for the reduction in the apparent dendrite coarsening parameter *A*. Ultimately, under conditions where the local solidification time remains essentially unchanged, this achieves a significant reduction in the spacing between secondary dendrite arms. This mechanism not only refines the theory of non-equilibrium solidification for multi-component Co-Al-W-based superalloys over a wide range of Co/Ni ratios, but also provides comprehensive theoretical support and quantitative data for the composition gradient design, precise control of casting processes, and optimization of subsequent heat treatment processes for high-performance alloys in this system.

## 5. Conclusions

This study systematically investigated the mechanisms by which the Co/Ni ratio in the range of 0.6–2.0 influences the solidification characteristics, dendrite morphology evolution, and element segregation behavior of Co-Al-W-based superalloy systems. It elucidated the thermodynamic–kinetic synergistic regulation mechanism underlying these phenomena. The main conclusions are as follows:

1. Adjusting the Co/Ni ratio can significantly broaden the alloy’s heat treatment window without compromising the stability of the casting process. In the multi-component system studied here, changes in the Co/Ni ratio had no significant effect on the liquidus and solidus temperatures of the alloy; the solidification temperature range remained stable at 87–111 °C, ensuring the stability of the casting process window. However, increasing the Co/Ni ratio significantly lowered the dissolution temperature of the γ′ phase, expanding the alloy’s heat treatment window from 215 °C to a maximum of 269 °C.
2. Increasing the Co/Ni ratio can significantly reduce the spacing between secondary dendrite arms by suppressing microsegregation of elements and lowering the apparent dendrite coarsening parameter *A*. When the Co/Ni ratio was increased from 0.6 to 2.0, the SDAS in the center of the ingot decreased from 112.4 μm to 43.3 μm, representing a reduction of 61.5%; normalized calculations show that the maximum reduction in the apparent dendrite coarsening parameter *A*. reaches 95.5%, with its theoretical contribution to the reduction in SDAS being approximately 64%. This is in close agreement with the experimental results, indicating that the apparent A parameter is a key factor reflecting the regulation of dendrite morphology by the Co/Ni ratio.
3. The kinetic mechanism by which the Co/Ni ratio suppresses microsegregation during solidification was elucidated. Adjusting the Co/Ni ratio does not alter the solid–liquid thermodynamic partition tendencies of elements such as Al, Ta, Hf, and W, but an increase in Co content can significantly reduce the diffusion activation energy of refractory elements, increase their mutual diffusion coefficients, and accelerate the reverse homogenizing diffusion of solute atoms during solidification. Combined with the depletion of elements prone to interdendritic segregation by TCP phase during the late stages of solidification, this causes the distribution coefficients of the major segregating elements to approach 1, thereby significantly improving the chemical homogeneity of the as-cast matrix.
4. A multidimensional synergistic regulation mechanism for the solidification behavior of Co-Al-W-based superalloys as a function of the Co/Ni ratio has been proposed. This mechanism clarifies how an increase in the Co/Ni ratio accelerates solute diffusion, thereby suppressing element segregation, reducing the driving force for dendrite coarsening, and ultimately leading to a refined as-cast dendritic microstructure.
5. Although this study systematically elucidates the non-equilibrium solidification behavior and as-cast microstructural evolution of Co-Al-W-based superalloys, the high-temperature mechanical properties of the alloys after complete heat treatment remain to be further explored. Future research will focus on optimizing the homogenization heat treatment process based on the widened heat treatment window and systematically evaluating the high-temperature creep and oxidation resistance of the fully heat-treated alloys, thereby providing comprehensive guidance for their engineering applications.

## Figures and Tables

**Table 1:** Measured and designed compositions of the investigated Co-Ni-based superalloys (wt.%) [36].

Alloys		Co	Ni	Fe	Al	W	Ta	Cr	Hf	C
0.6Co/Ni	Designed	Bal.	37.50	4.00	5.00	11.00	7.50	12.00	0.20	0.05
	Measured	Bal.	37.40	4.06	4.96	10.78	7.33	12.59	0.19	0.04
1.2Co/Ni	Designed	Bal.	27.30	4.00	5.00	11.00	7.50	12.00	0.20	0.05
	Measured	Bal.	27.35	3.97	4.98	11.09	7.28	11.98	0.21	0.04
1.5Co/Ni	Designed	Bal.	24.00	4.00	5.00	11.00	7.50	12.00	0.20	0.05
	Measured	Bal.	24.23	3.98	4.96	11.08	7.33	11.93	0.19	0.04
2.0Co/Ni	Designed	Bal.	20.00	4.00	5.00	11.00	7.50	12.00	0.20	0.05
	Measured	Bal.	20.38	3.99	4.98	11.05	7.23	11.82	0.20	0.04

**Table 2:** Solidification Characteristic Temperatures of Alloys with Different Co/Ni Ratios Measured by DSC.

Alloy	Solidification Characteristic Temperatures/°C				
	*T*_L_	*T*_s_	*T*_γ′_	*HTW*	Δ*T*
0.6Co/Ni	1345	1258	1043	215	87
1.2Co/Ni	1348	1237	1017	220	111
1.5Co/Ni	1346	1243	974	269	103
2.0Co/Ni	1352	1241	/	/	111

**Table 3:** Secondary dendrite arm spacing (SDAS) in different regions of the cross-section of ingots made from alloys with varying Co/Ni ratios.

Alloys	SDAS at Different Radial Positions (μm)		
	Center	R/2	Edge
0.6Co/Ni	112.4 ± 2.5	52.3 ± 2.0	42.7 ± 1.5
1.2Co/Ni	89.3 ± 1.4	66.2 ± 2.1	47.2 ± 1.6
1.5Co/Ni	47.5 ± 1.1	37.1 ± 1.3	32.5 ± 0.9
2.0Co/Ni	43.3 ± 1.4	35.7 ± 1.1	31.4 ± 0.9

**Table 4:** Solid–Liquid Fractionation Coefficients of Major Elements in Four Alloys with Different Co/Ni Ratios Based on J-Matpro Simulation.

Alloy	Initial Partition Coefficient ($p\;={\;C}_{S}/C_{L}$)				
	*p*_Al_	*p*_W_	*p*_Ta_	*p*_Hf_	*p*_Cr_
0.6Co/Ni	0.86	1.15	0.23	0.90	1.02
1.2Co/Ni	0.84	1.22	0.17	0.91	1.05
1.5Co/Ni	0.83	1.24	0.16	0.91	1.06
2.0Co/Ni	0.82	1.26	0.15	0.91	1.07

**Table 5:** Dendritic segregation coefficient *K*_i_ of as-cast microstructure in four groups of alloys with different Co to Ni ratios based on EPMA measurements.

Alloy	Partitioning Ratio (*K*_i_$\;=\;C_{\mathrm{ID}}^{i}/C_{\mathrm{DC}}^{i}$)							
	*K*_Ni_	*K*_Co_	*K*_Al_	*K*_W_	*K*_Ta_	*K*_Cr_	*K*_Fe_	*K*_Hf_
0.6Co/Ni	1.01 ± 0.03	0.92 ± 0.04	1.20 ± 0.07	0.75 ± 0.05	1.93 ± 0.14	1.03 ± 0.04	0.88 ± 0.05	1.50 ± 0.11
1.2Co/Ni	0.98 ± 0.02	0.96 ± 0.03	1.11 ± 0.06	0.84 ± 0.04	1.73 ± 0.11	1.05 ± 0.03	0.87 ± 0.04	1.32 ± 0.08
1.5Co/Ni	1.01 ± 0.03	0.96 ± 0.02	1.06 ± 0.04	0.89 ± 0.03	1.50 ± 0.09	1.01 ± 0.02	0.92 ± 0.03	1.14 ± 0.06
2.0Co/Ni	0.94 ± 0.02	0.96 ± 0.03	1.02 ± 0.03	0.93 ± 0.04	1.27 ± 0.07	1.02 ± 0.03	0.90 ± 0.04	1.06 ± 0.05

**Table 6:** Normalized results of the apparent dendrite coarsening parameter *A* in the central region of ingots made from alloys with different Co/Ni ratios.

Alloy	Co/Ni Ratio	SDAS λ_2_ (μm)	ΔT (°C)	Relative Value A (0.6Co/Ni = 1)	Relative Decline in A
0.6Co/Ni	0.6	112.4	87	1.000	0%
1.2Co/Ni	1.2	89.3	111	0.393	60.7%
1.5Co/Ni	1.5	47.5	103	0.064	93.6%
2.0Co/Ni	2.0	43.3	111	0.045	95.5%

## Data Availability

The original contributions of this study are included in the article. Further inquiries can be directed to the corresponding author.
